# Prevalence of Cyberchondria among Outpatients with Metabolic Syndrome in a Tertiary Care Hospital in Southern India

**DOI:** 10.1155/2022/3211501

**Published:** 2022-09-26

**Authors:** Priya Pawar, Ashok Kamat, Gavishiddhayya Salimath, Kiran Rose Jacob, Rajesh Kamath

**Affiliations:** ^1^Department of Psychiatric Nursing, KAHER Institute of Nursing Sciences, KLE University, Belgaum, India; ^2^Department of Child Health and Nursing, KAHER Institute of Nursing Sciences, KLE University, Belgaum, India; ^3^Prasanna School of Public Health, Manipal Academy of Higher Education, Manipal, India

## Abstract

**Background:**

In today's world, Internet-based medical information plays a significant role in patient education. There are several accessible health-related websites. It has become common to search Internet before going for a medical consultation. The main objective of this study was to determine the prevalence of cyberchondriasis and its association with demographic variables.

**Methods:**

A cross-sectional study was carried out among metabolic syndrome patients attending the cardiology, endocrinology, and neurology outpatient departments of a tertiary care hospital in South India. The prevalence of cyberchondriasis and its constructs were measured using the cyberchondria severity scale (CSS). Inferential statistics revealed no statistically significant difference in the average CSS scores across sociodemographic variables. Spearman correlation was conducted to determine the relationship between the constructs.

**Results:**

A total of 379 participants with metabolic syndrome were included in the study. 42.5% of them were severely affected, and 28.0% were moderately affected by cyberchondriasis. Among the constructs studied, compulsion (85.7%), distress (91.8%), excessiveness (96.6%), and reassurance (76.1%) constructs had an impact on a greater number of study participants, compared to mistrust of medical professional construct (33.0%). Cyberchondriasis had a significant relationship with the history of myocardial infarction (*p* value = 0.03). There was a statistically significant positive linear relationship between mistrust and reassurance (*r*_*s*_ = 0.169, *p* value<0.001). Reassurance had a significant negative linear relationship with distress (*r*_*s*_ = −0.147, *p* value = 0.004).

**Conclusion:**

In India, cyberchondriasis is a growing public mental health issue. Awareness among the general population is necessary to minimize the possible outcomes of cyberchondriasis like anxiety and depression. Screening individuals for possible risk factors is recommended.

## 1. Introduction

In today's world, Internet-based medical information plays a significant role in patient education. The use of Internet as a source of health information has rapidly increased [1, 2]. Internet has become a popular means of accessing health information because it is quick, easy to use, anonymous, and relatively inexpensive [3].

Using Internet for health information can lead to misdiagnosis and exploitation [1, 4, 5]. It has become common to search Internet before going for a medical consultation. People find information that drives them into anxiety and distress. This phenomenon is referred to as cyberchondria.

Cyberchondria consists of four core dimensions: Internet searches for medical information repetitively (excessiveness); increased negative affect (distress); interrupted daily living (compulsion); and engendered reassurance seeking. Individuals tend to develop disorders such as health-related anxiety and depression in response to the information obtained from online sources [6]. More than half of the walk-in patients in a hospital had significant levels of depressive symptoms and had searched Internet for information about their health concerns [7]. Cyberchondria increases the concern about differentiating between reliable and unreliable online sources of information [3]. Peer or professional reviews, personal blogs, opinions, or experiences from other patients can be obtained from Internet. The quality of information varies, and clients may possess the ability to evaluate medical information and link it to their health situations [8]. 67% of physicians reported dealing with patients who talked about their medical conditions based on their information from online resources [9]. People with cyberchondria have differentiated characteristics regarding their actions and thoughts, including spending hours daily checking their symptoms online. People with higher illness anxiety spent more time on Internet, whereas people with mild health anxiety spent relatively less time in a day. With an existing fear of contracting diseases, online medical consultation makes these individuals more anxious about their health. People suffering from high anxiety are more likely to exaggerate their perceived disability [10]. One source of information is rarely enough for them, and so, they refer to at least two or more websites at a time, which can lead to physical symptoms like worsening anxiety, increased heartbeat, difficulty in breathing, and tightness of the throat. The more emotionally overwhelmed one feels, the more they are likely to spend time searching for the particular symptom, and the more they search, the more they are convinced that they are experiencing a certain illness. At this stage, an individual is more likely to begin to trust the Internet and mistrust the physician, thus displaying a common attribute of cyberchondria [11]. One of the other significant consequences of the excessive online search for health-related information can be self-medication [12].

Searching Internet before going for a medical consultation is a growing trend in India. Although some evidence suggests that individuals with high levels of health anxiety may seek health information online more frequently and that this behaviour could fuel health anxiety, this is yet to be explicitly examined. The current study was conducted to determine the prevalence of cyberchondriasis among patients with metabolic syndrome.

## 2. Methods

A descriptive cross-sectional survey was conducted in a tertiary care hospital in South India from February 2021 to March 2021. Ethical clearance was obtained from the institutional ethical committee and informed consent was obtained from participants. 379 respondents participated in the study. The cyberchondria severity scale (CSS) questionnaire was used to collect details regarding the health information obtained from Internet. The patients who were aged 30 years and above, had metabolic syndrome conditions, and were literate were included in the study. Patients who were aged below 30 years, did not have metabolic syndrome conditions, were not literate, and were severely ill were excluded from the study. Data were analyzed using descriptive and inferential statistics.

The sociodemographic information included age, gender, education, religion, type of family, family income, and use of Internet (Table 1). The prevalence of chronic conditions among participants was also included (Table 2). The cyberchondria severity scale (CSS)-15 was used to assess the prevalence of cyberchondriasis (Table 3). The scale comprises 15 items, categorized into five subscales with three questions in each. These subscales are compulsiveness (CM), distress (DS), excessiveness (EX), reassurance (RE), and mistrust (MS). Subscales were used to determine the prevalence of the constructs with each item rated on a 4-point scale (0-never, 1-rarely, 2-sometimes, 3-often, and 4-always). If the score was between 1 and 6, the participant was considered “moderately affected”; if the score was 7 or above, the participant was considered “highly affected.” If the score was 0, the participant was considered “not affected” [12]. Cyberchondriasis was calculated by adding the scores, and if the total was below 37, the participant was considered less affected. The participant was considered moderately affected if it was between 37 and 40. If it was above 40, it was categorized as severely affected. Nonparametric inferential methods (Mann–Whitney *U* test and Kruskal–Wallis ANOVA) were carried out to observe whether there is a statistically significant difference in the average CSS scores across sociodemographic variables. Spearman's correlation was conducted to determine the relationship between the constructs.

## 3. Results

As depicted in Table 4, based on the Mann–Whitney *U* test, at a 5% level of significance, there was a statistically significant difference in the average cyberchondriasis score across the history of myocardial infarction (*p* value = 0.03). Furthermore, the prevalence of individual constructs showed that every participant was severely or moderately affected by at least one aspect of CSS. Thus, no significant association was noticed between the overall CSS score and sociodemographic variables.

Spearman correlation analysis revealed a statistically significant positive linear relationship between mistrust and reassurance (*r*_*s*_ = 0.169, *p* value <0.001, *α* = 5%). Reassurance has a significant negative linear relationship with distress (*r*_*s*_ = −0.147, *p* value = 0.004).

## 4. Discussion

The study's overall results indicate that the participants were affected by the five constructs (to a varying extent) after searching for online health information. Among all the constructs studied, as depicted in Table 5, compulsion (85.7%), distress (91.8%), excessiveness (96.6%), and reassurance (76.1%) constructs had an impact on a more significant number of study participants than the mistrust of medical professionals (33.0%).

This study's findings on the constructs of cyberchondria are similar to a recent Indian study in which it was found that all the participants were affected by excessiveness [13]. These results are similar to another study conducted to examine the relationship between health anxiety and searching for health information on Internet [14]. The reassurance construct influenced 92%, and the literature shows that as a cyberchondriac searches Internet for a second or third opinion, they start to identify themselves with the most serious cases [15]. The present study found that the distress constructs affected 91.8% of the participants, similar to a study conducted to examine the positive correlation between anxiety and searching for illness and wellness information [11]. 75% of the participants were affected by compulsion, and similar results were obtained in a few other studies conducted [3, 16]. 33% were influenced by mistrust of the medical professional. This, however, is strikingly higher than what was found in another study that examined the prevalence of cyberchondria in graduate employees [13]. This result is supported by the study conducted to understand the role of the Internet in patient-practitioner relationships, which reported that the patients are more confident with the information given by their doctor than on Internet [1].

The findings suggest that using Internet for health information is likely to develop anxiety in some people, to a point where their Internet use begins to feel excessive, out of control, and symptomatic of their broader difficulties. This behaviour may promote self-medication, considering that this type of information can also be shown explicitly on Internet sites [3]. Compared to other health information sources (e.g., medical textbooks and health information leaflets), Internet contains much-unregulated health information. Search engine architects are responsible for ensuring that searchers do not experience unnecessary concerns generated by the definitions of relevance and the ranking algorithms their engines use. Because people may believe they can conduct their diagnosis based on search results, Internet searching habit may impact and delay professional help. This information explains a previously unknown aspect of the doctor-patient relationship. Doctors will need more patience and time to interact with Internet-literate patients, reassuring them about their search and determining whether the information they have found is correct or not. Future search engines should incorporate ranking algorithms in medical domains [17], iterative intelligent medical search engines [18], and classifiers to indicate when a user is using a search engine as a probabilistic diagnostic system [18], such that accurate base rates of illness are more accessible to symptom-searchers on Internet. Those with illness worries may consider installing blocking software to prevent anxiety-provoking health searches, as such an approach has been effective for another pathological checking [10].

To some extent, search results from scientific and trustworthy sites provide accurate information; however, if people are surfing lay forums, there is cause for concern for both the patient and the doctor. There is scope for further research to understand how health-related Internet use can be harmful to anxious individuals. Increasing health literacy is another method to reduce Internet-related escalations of distress among those with illness anxiety, as it will help differentiate reliable and unreliable information.

### 4.1. Limitation

Only patients attending cardiology, endocrinology, and neurology outpatient departments in a single tertiary care hospital were included in the study. Thus, the results cannot be generalized.

### 4.2. Main Messages

- In India, cyberchondria is a growing public mental health issue that needs immediate attention or can cause significant harm to the general population
- To get a better understanding of the prevalence of cyberchondria and the factors influencing it, there should be more research conducted in this field
- Both prevention and treatment measures should be taken by the authorities on a community level to reduce the risk of cyberchondria

### 4.3. What Is Already Known on the Subject

- Cyberchondria is a form of anxiety characterized by excessive online health research
- Cyberchondria consists of four core dimensions: Internet searches for medical information repetitively (excessiveness); increased negative affect (distress); interrupted daily living (compulsion); and engendered reassurance seeking
- People with cyberchondria have differentiated characteristics in terms of their actions and thoughts, including spending hours daily checking their symptoms online

## Figures and Tables

**Table 1:** Distribution of sociodemographic characteristics of the participants.

Variable	Category	Count (%)
Age in year	Up to 60 years	292 (77.0)
	Above 60 years	87 (23.0)

Gender	Male	186 (49.1)
	Female	193 (50.9)

Education	Formal education	170 (44.9)
	Graduation and above	209 (55.1)

Religion	Hindu	104 (27.4)
	Christian	160 (42.2)
	Muslim	85 (22.4)
	Others	30 (7.9)

Types of family	Nuclear	189 (49.9)
	Joint	190 (50.1)

Income	Below 20000	185 (48.8)
	20000 and more	194 (51.2)

Marital status	Living with spouse	347 (91.6)
	Living without spouse	32 (8.4)

Usage of Internet	Below 3 hours	94 (24.8)
	Above 3 hours	285 (75.2)

**Table 2:** History of chronic conditions among the participants.

Condition	Count (%)
Hypertension	257 (67.8)
Diabetes mellitus	244 (64.4)
Myocardial infarction	25 (6.6)
Stroke	25 (6.6)

**Table 3:** Prevalence of cyberchondria among the participants.

Components	Count (%)
Severely affected	161 (42.5)
Moderately affected	106 (28.0)
Less affected	112 (29.6)

**Table 4:** Association between cyberchondriasis severity scale and various factors.

Variable	Category	Median	Interquartile range	*P* value
Age in year^#^	Up to 60 years	39.00	5.00	0.68
	Above 60 years	39.00	6.00	

Gender^#^	Male	39.00	5.00	0.87
	Female	39.00	7.00	

Education^#^	Formal education	39.00	5.75	0.13
	Graduation and above	38.00	6.00	

Religion^+^	Hindu	38.00	5.00	0.95
	Christian	39.00	5.00	
	Muslim	39.00	6.00	
	Others	38.50	8.50	

Types of family^#^	Nuclear	38.50	6.00	0.57
	Joint	39.00	5.00	

Income^#^	Below 20000	39.00	6.00	0.37
	20000 and above	39.00	6.00	

Marital status^#^	Living with spouse	39.00	5.50	0.46
	Living without spouse	38.50	5.50	

Usage of Internet^#^	Below 3 hours	39.00	5.00	0.75
	3 hours and above	39.00	6.00	

Hypertension^#^	Yes	39.00	5.00	0.67
	No	39.00	6.00	

Diabetes mellitus^#^	Yes	39.00	6.00	0.42
	No	39.00	5.00	

Myocardial infarction^#^	Yes	36.00	6.00	0.03^*∗*^
	No	39.00	6.00	

Stroke^#^	Yes	36.00	5.00	0.11
	No	39.00	6.00	

^*∗*^Significant at *α* = 5%. ^#^Findings from the Mann–Whitney *U* test. ^+^Findings from the Kruskal–Wallis test.

**Table 5:** Prevalence of different constructs of cyberchondria.

Components	Count (%)
Level of compulsiveness	
Severely affected	323 (85.7)
Moderately affected	54 (14.3)
Not affected	0 (0.0)

Level of distress	
Severely affected	346 (91.8)
Moderately affected	31 (8.2)
Not affected	0 (0.0)

Level of exclusiveness	
Severely affected	364 (96.6)
Moderately affected	13 (3.4)
Not affected	0 (0.0)

Level of reassurance	
Severely affected	287 (76.1)
Moderately affected	90 (23.9)
Not affected	0 (0.0)

Level of mistrust	
Severely affected	124 (33.0)
Moderately affected	252 (67.0)
Not affected	0 (0.0)

## Data Availability

The data used to support the findings of this study are available from the corresponding author upon request.
